# Supplementary material for: Evidence for protection of targeted reef fish on the largest marine reserve in the Caribbean
Source: PeerJ. 2014 Feb 20;2:e274. doi: 10.7717/peerj.274 (PMC3932734; doi:10.7717/peerj.274)
Supplement: Supplemental Information 1 — Names and coordinates (dd, mm, ss) of the study sites by zones and reef habitat type (slope and crest). Depth range for reef slopes was 12-15m while for reef crest was 2-3m. Sites without coordinate values were not sampled in that habitat. [file peerj-02-274-s001.docx]

Table S1

|  |  | **Reef Slope** | | **Reef Crest** | |
| --- | --- | --- | --- | --- | --- |
| Zones | Site Names | Latitude | Longitude | Latitude | Longitude |
| No Reserve West (NRW) | Bretón | 21,03,52 | 79,26,12 | 21,04,03 | 79,25,29 |
|  | Horqueta | 21,04,02 | 79,23,36 | 21,04,16 | 79,23,57 |
|  | Alcatraz | 21,02,54 | 79,20,24 | 21,02,83 | 79,20,28 |
|  | Alcatraz 2 | 21,02,05 | 79,19,88 | - | - |
|  | Cinco Balas | 21,01,45 | 79,17,34 | 21,01,51 | 79,17,44 |
| Reserve West (RW) | Boca de Guano | 20,57,49 | 79,10,45 | 20,58,00 | 79,10,47 |
|  | Caseta Blanca | 20,55,38 | 79,07,58 | 20,55,48 | 79,08,06 |
|  | Caseta Blanca | 20,55,01 | 79,05,48 | - | - |
|  | Punta Escondida | 20,51,44 | 79,03,30 | 20,51,26 | 79,02,53 |
|  | Los Pinos | 20,50,61 | 79,00,71 | 20,50,76 | 79,00,78 |
| Reserve Center (RC) | La Puntica | - | - | 20,49,55 | 78,58,52 |
|  | El Partido | 20,46,97 | 78,55,74 | - | - |
|  | Mariflores | 20,46,14 | 78,53,48 | 20,46,30 | 78,53,61 |
|  | Boca de Piedra | 20,45,70 | 78,50,62 | 20,45,72 | 78,50,65 |
|  | Piedra Grande | 20,44,08 | 78,48,59 | 20,43,63 | 78,47,81 |
|  | Las Cruces | 20,42,33 | 78,46,59 | - | - |
| Reserve East (RE) | Cachiboca | 20,40,08 | 78,44,45 | - | - |
|  | Ballena | 20,38,56 | 78,41,09 | - | - |
|  | Carabinero | 20,38,39 | 78,39,51 | - | - |
|  | Carabinero 2 | 20,38,18 | 78,38,47 | - | - |
|  | Bártolo | 20,38,08 | 78,37,41 | - | - |
| No Reserve East (NRE) | Boca Seca | 20,36,46 | 78,32,57 | - | - |
|  | Peralta | 20,35,56 | 78,29,10 | - | - |
|  | Punta Macao | 20,33,11 | 78,24,57 | - | - |
|  | Cabeza del Este | 20,31,30 | 78,23,38 | - | - |
|  | Cabeza del Este 2 | 20,29,92 | 78,22,03 | - | - |
