# Supplementary material for: Evidence for protection of targeted reef fish on the largest marine reserve in the Caribbean
Source: PeerJ. 2014 Feb 20;2:e274. doi: 10.7717/peerj.274 (PMC3932734; doi:10.7717/peerj.274)
Supplement: Supplemental Information 2 — Common names are organized in the same order as in Table 1. [file peerj-02-274-s002.docx]

Table S2

| Common Name | Scientific Name | Family |
| --- | --- | --- |
| Nassau grouper | *Epinephelus striatus* | Serranidae |
| Hogfish | *Lachnolaimus maximus* | Labridae |
| Schoolmaster | *Lutjanus apodus* | Lutjanidae |
| Cubera snapper | *L. cyanopterus* | Lutjanidae |
| Dog snapper | *L. jocu* | Lutjanidae |
| Mutton snapper | *L. analis* | Lutjanidae |
| Yellowfin grouper | *Mycteroperca venenosa* | Serranidae |
| Tiger grouper | *M. tigris* | Serranidae |
| Black grouper | *M. bonaci* | Serranidae |
| Great barracuda | *Sphyraena barracuda* | Sphyraenidae |
| Spotted eagle ray | *Aetobatus narinari* | Myliobatidae |
| Yellow jack | *Caranx bartholomaei* | Carangidae |
| Crevalle jack | *C. hippos* | Carangidae |
| Horse-Eye jack | *C. latus* | Carangidae |
| Reef shark | *Carcharhinus perezi* | [Carcharhinidae](http://www.fishbase.org/summary/FamilySummary.php?ID=11) |
| Silky shark | *C. falciformis* | [Carcharhinidae](http://www.fishbase.org/summary/FamilySummary.php?ID=11) |
| Southern stingray | *Dasyatis americana* | Dasyatidae |
| Goliath grouper | *Epinephelus itajara* | Serranidae |
| Nurse shark | *Ginglymostoma cirratum* | [Ginglymostomatidae](http://www.fishbase.org/summary/FamilySummary.php?ID=493) |
| Margate | *Haemulon album* | Haemulidae |
| Tarpon | *Megalops atlanticus* | Megalopidae |
| Lemon shark | *Negaprion brevirostris* | [Carcharhinidae](http://www.fishbase.org/summary/FamilySummary.php?ID=11) |
| Rainbow parrotfish | *Scarus guacamaia* | Scaridae |
| Midnight parrotfish | *S. coelestinus* | Scaridae |
| King mackerel | *Scomberomorus cavalla* | Scombridae |
| Spanish mackerel | *S. maculatus* | Scombridae |
| Cero | *S. regalis* | Scombridae |
| Permit | *Trachinotus falcatus* | Carangidae |
