# Supplementary material for: Evidence for protection of targeted reef fish on the largest marine reserve in the Caribbean
Source: PeerJ. 2014 Feb 20;2:e274. doi: 10.7717/peerj.274 (PMC3932734; doi:10.7717/peerj.274)
Supplement: Supplemental Information 3 — Numbers are mean standard error. Time is the month and year of survey for algae assemblages. Superscript letters represent significant differences among zones within the same reef habitat after a one-way ANOVA and a Student-Newman-Keuls post hoc analysis (letters: p < 0.05, no letters: p > 0.05). No statistical tests were performed on % cover of algae functional groups. [file peerj-02-274-s003.docx]

Table S3

| **Variable** | **Time** | **Reef Slope** | | | | | **Reef Crest** | | |
| --- | --- | --- | --- | --- | --- | --- | --- | --- | --- |
|  |  | NRW | RW | RC | RE | NRE | NRW | RW | RC |
| Reef structural complexity |  | 4.2±0.4 | 4.3±0.3 | 4.5±0.2 | 4.3±0.2 | 4.3±0.4 | 4.7±0.2 | 4.6±0.2 | 4.4±0.4 |
| CORALS |  |  |  |  |  |  |  |  |  |
| Coral density (col/m^2^) |  | 17.8±1.0 | 17.2±1.3 | 18.5±0.7 | 16.3±1.6 | 18.1±1.0 | 6.7±0.6 | 6.8±0.7 | 6.2±0.4 |
| Massive |  | 0.11±0.02^a^ | 0.13±0.01^a^ | 0.19±0.02^b^ | 0.11±0.02^a^ | 0.06±0.01^a^ | 0.04±0.01 | 0.05±0.01 | 0.08±0.02 |
| Plating |  | 1.2±0.2^a^ | 1.2±0.1^b^ | 1.2±1.1^b^ | 1.1±0.2^b^ | 1.6±0.2^b^ | 0.4±0.1 | 0.5±0.1 | 0.3±0.1 |
| Encrusting |  | 0.6±0.03^b^ | 0.5±0.03^b^ | 0.6±0.02^a^ | 0.5±0.04^b^ | 0.5±0.02^b^ | 0.2±0.02 | 0.2±0.03 | 0.2±0.02 |
| Branching |  | 0.4±0.03 | 0.3±0.06 | 0.3±0.02 | 0.3±0.04 | 0.3±0.05 | 0.3±0.04 | 0.2±0.03 | 0.2±0.02 |
| Recruits density (col/m^2^) |  | 1.6±0.1^a^ | 0.9±0.12^b^ | 1.6±0.2^a^ | 0.8±0.16^b^ | 0.9±0.04^b^ | 0.2±0.11 | 0.3±0.08 | 0.1±0.03 |
| Coral cover (%) |  | 15.8±1.0 | 18.7±1.0 | 18.0±1.0 | 16.0±1.7 | 17.1±1.0 | 11.8±1.9 | 8.4±2.2 | 6.8±1.0 |
| Bleaching (%) |  | 1.9±0.4 | 1.4±0.2 | 1.4±0.4 | 1.4±0.5 | 2.0±0.2 | 0.7±0.2 | 0.8±0.3 | 0.2±0.1 |
| Mortality (%) |  | 7.0±0.8 | 8.3±0.7 | 5.6±1.3 | 8.2±0.8 | 7.4±1.3 | 19.1±2.2 | 21.5±2.3 | 22.6±0.6 |
| Richness (S) |  | 24.8±1.0 | 24.1±0.9 | 25.0±1.1 | 24.8±1.0 | 24.0±1.0 | 14.6±0.8^a^ | 11.5±0.7^b^ | 13.2±1.0^ab^ |
| Evenness (J) |  | 0.7±0.02^ab^ | 0.8±0.01^a^ | 0.8±0.02^a^ | 0.8±0.03^ab^ | 0.8±0.02^b^ | 0.8±0.02 | 0.8±0.05 | 0.7±0.06 |
| Diversity (H) |  | 2.3±0.1^a^ | 2.5±0.1^ab^ | 2.5±0.1^ab^ | 2.5±0.1^ab^ | 2.6±0.1^b^ | 2.0±0.1 | 1.9±0.1 | 1.8±0.1 |
| Maximum size (Ø cm) |  | 15.5±0.6 | 18.2±0.4 | 15.6±0.7 | 18.9±1.3 | 17.6±1.0 | 37.7±1.8 | 41.2±2.3 | 39.5±1.6 |
| ALGAE (% cover) |  |  |  |  |  |  |  |  |  |
| Total Algae |  | 70.8±7.1 | 69.1±9.5 | 69.0±9.7 | 61.9±8.8 | 67.6±14.8 | 59.5±0.6 | 60.3±4.2 | 62.5±2.2 |
| Foliose | Jun 04 | 35.5±3.3 | 14.7±1.7 | 14.6±1.7 | 14.8±2.4 | 14.7±2.0 | 0.1±0.1 | 0.3±0.2 | 1.2±0.6 |
|  | Jan 05 | 35.2±2.7 | 30.3±2.4 | 29.3±2.8 | 24.2±1.5 | 31.9±2.6 | 1.7±0.7 | 0.8±0.4 | 0.3±0.3 |
|  | Apr 05 | 38.2±2.6 | 30.3±2.4 | 28.2±2.8 | 26.4±2.6 | 39.8±2.6 | 1.1±0.5 | 2.1±1.0 | 0.3±0.3 |
| Filamentous | Jun 04 | 6.3±1.6 | 2.5±0.9 | 8.9±1.9 | 3.1±1.0 | 9.8±1.8 | 20.8±3.7 | 9.1±1.3 | 13.5±3.7 |
|  | Jan 05 | 9.8±1.6 | 5.3±1.3 | 10.8±2.5 | 17.4±1.4 | 20.4±2.1 | 5.9±1.4 | 13.6±1.9 | 13.0±2.8 |
|  | Apr 05 | 9.5±1.7 | 5.3±1.3 | 8.9±1.7 | 6.0±1.4 | 14.1±1.4 | 6.1±1.4 | 11.0±1.6 | 13.0±2.8 |
| Corticated | Jun 04 | 0.0 | 0.0 | 0.2±0.2 | 0.1±0.1 | 0.0 | 0.2±0.1 | 0.3±0.2 | 1.1±0.4 |
|  | Jan 05 | 0.0 | 0.0 | 0.0 | 1.3±0.5 | 1.2±0.7 | 0.1±0.1 | 0.5±0.2 | 2.2±1.0 |
|  | Apr 05 | 0.0 | 0.0 | 0.0 | 0.0 | 0.1±0.1 | 0.1±0.1 | 0.4±0.2 | 2.2±1.0 |
| Coriaceous | Jun 04 | 6.9±2.0 | 23.5±2.4 | 16.9±1.9 | 11.6±2.1 | 5.5±1.1 | 0.0 | 0.0 | 0.1±0.1 |
|  | Jan 05 | 19.4±2.7 | 28.8±2.4 | 24.9±3.0 | 13.5±1.9 | 18.4±2.1 | 0.0 | 0.6±0.3 | 0.5±0.3 |
|  | Apr 05 | 20.5±2.7 | 28.7±2.3 | 28.0±2.7 | 15.3±2.5 | 12.9±1.3 | 0.0 | 0.3±0.2 | 0.5±0.3 |
| Articulated calcareous | Jun 04 | 6.4±0.8 | 6.8±1.1 | 6.1±0.7 | 10.7±1.4 | 6.5±0.9 | 5.4±1.0 | 11.2±1.8 | 14.0±3.1 |
|  | Jan 05 | 8.1±1.2 | 11.7±1.0 | 8.2±1.1 | 12.7±1.3 | 10.6±1.2 | 5.8±1.3 | 8.1±1.9 | 9.0±2.2 |
|  | Apr 05 | 8.8±1.5 | 11.4±1.0 | 9.7±1.3 | 9.2±1.2 | 5.6±0.7 | 6.6±1.4 | 6.9±1.8 | 9.0±2.2 |
| Encrusting | Jun 04 | 1.8±1.0 | 2.5±0.5 | 2.9±0.6 | 5.3±1.4 | 2.4±0.8 | 31.9±3.1 | 31.5±3.3 | 28.2±5.0 |
|  | Jan 05 | 3.0±0.4 | 2.7±0.4 | 6.3±1.3 | 6.5±0.8 | 5.9±0.9 | 46.7±2.5 | 38.5±3.1 | 39.7±5.0 |
|  | Apr 05 | 3.0±0.4 | 2.7±0.4 | 3.2±0.8 | 7.7±1.2 | 3.0±0.6 | 46.0±2.7 | 45.8±3.2 | 39.7±5.0 |
