# Supplementary material for: Evidence for protection of targeted reef fish on the largest marine reserve in the Caribbean
Source: PeerJ. 2014 Feb 20;2:e274. doi: 10.7717/peerj.274 (PMC3932734; doi:10.7717/peerj.274)
Supplement: Supplemental Information 4 — Transformations are for each trophy species by reef habitat. Analyses were performed on the transformed data, figures are based on the raw data. [file peerj-02-274-s004.docx]

Table S4 Transformations needed for the two-way ANOVA

| **Species/groups** | **Reef slope x Time** | | | | **Reef crest x Time** |
| --- | --- | --- | --- | --- | --- |
| Black grouper | | Square root | | | Fourth root |
| Yellowfin grouper | | Square root | | | Square root |
| Tiger grouper | | Square root | | | Square root |
| Schoolmaster | | Fourth root | | | Fourth root |
| Nassau grouper | | Fourth root | | | Square root |
| Cubera snapper | | Fourth root | | | Square root |
| Dog snapper | | Natural log | | | Square root |
| Mutton snapper | | Fourth root | | | Fourth root |
| Hogfish | | Fourth root | | | Fourth root |
| Great barracuda | | Square root | | | Fourth root |
| Total trophy | | Square root | | | No transformation |
| Total trophy _(no schoolmaster)_ | | Square root | | | No transformation |
|  | |  |  |  |  |
