# Supplementary figures and images for: Evidence for protection of targeted reef fish on the largest marine reserve in the Caribbean
Source: PeerJ. 2014 Feb 20;2:e274. doi: 10.7717/peerj.274 (PMC3932734; doi:10.7717/peerj.274)

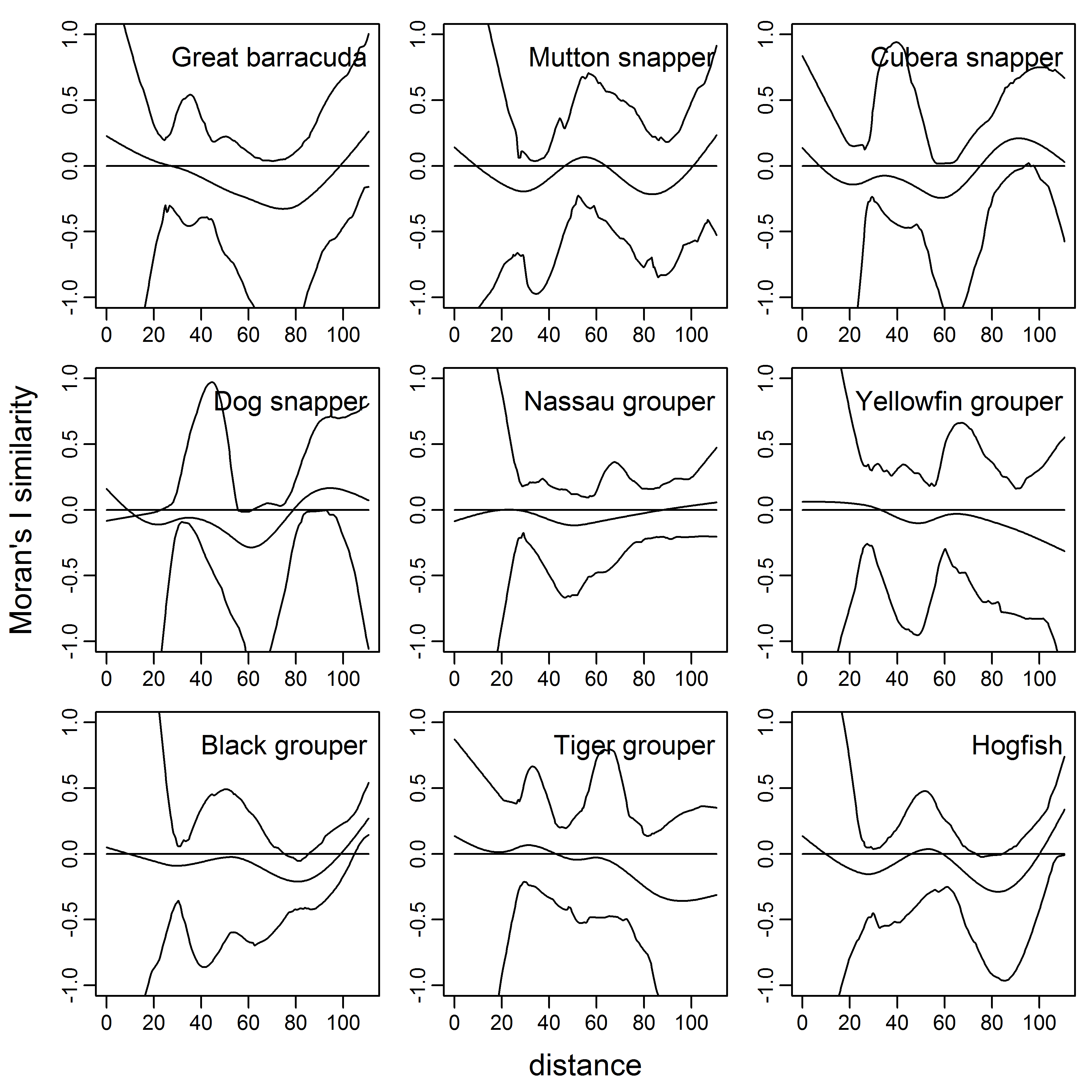

Supplement: Supplemental Information 5 — Spline correlograms using Moran’s I similarity index and lag distance in kilometers showing the lack of spatial autocorrelation of the model residuals for each of the nine trophy species analyzed in the slope reef habitat. Lines are mean (middle line) and 95% confidence interval (outer lines). Horizontal line is zero correlation. [file peerj-02-274-s005.png]

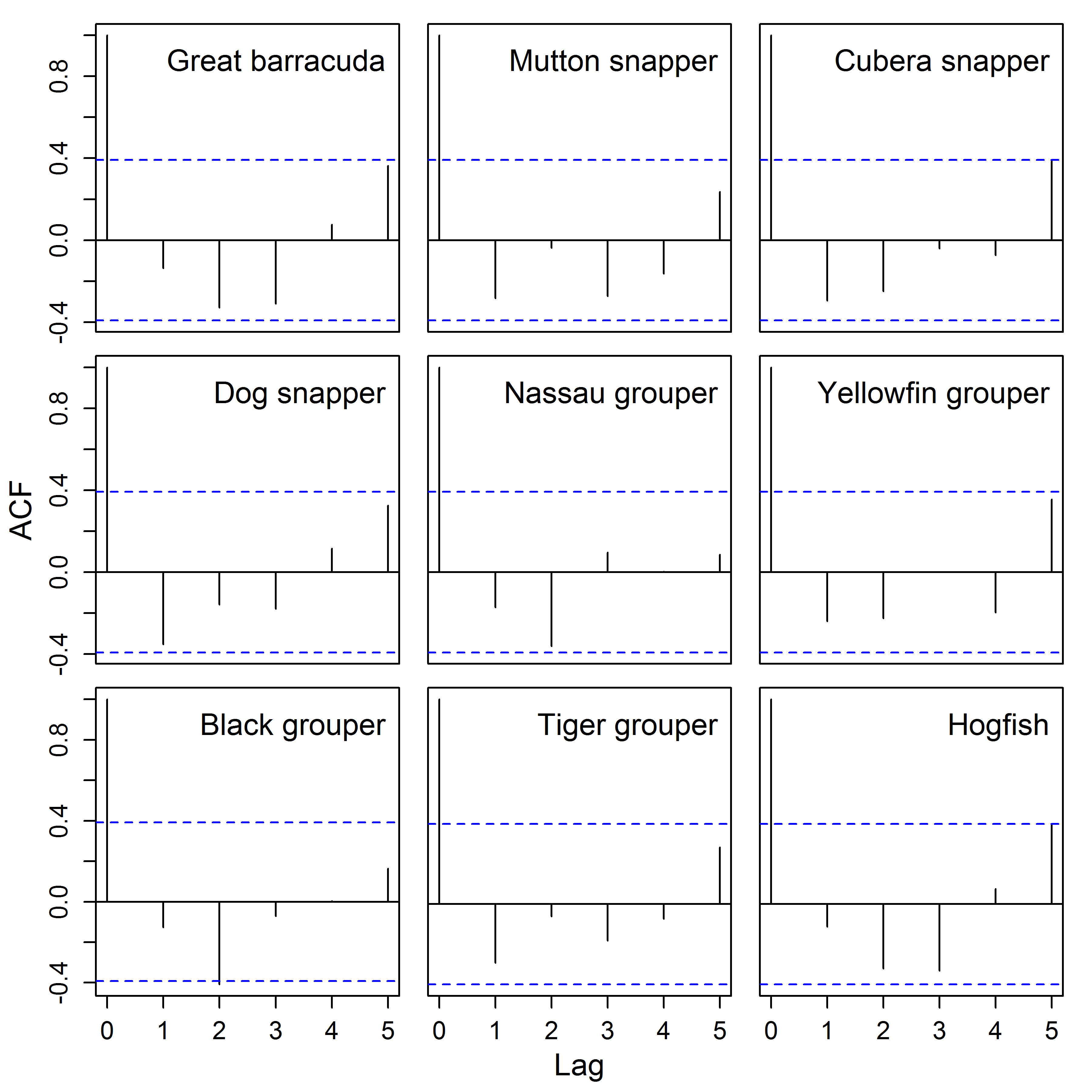

Supplement: Supplemental Information 6 — Autocorrelation function (ACF) values showing the lack of temporal correlation among five months (lag) for the nine trophy species in the reef slope habitat that showed significant interactions between zones and times based on the factorial ANOVA. [file peerj-02-274-s006.png]

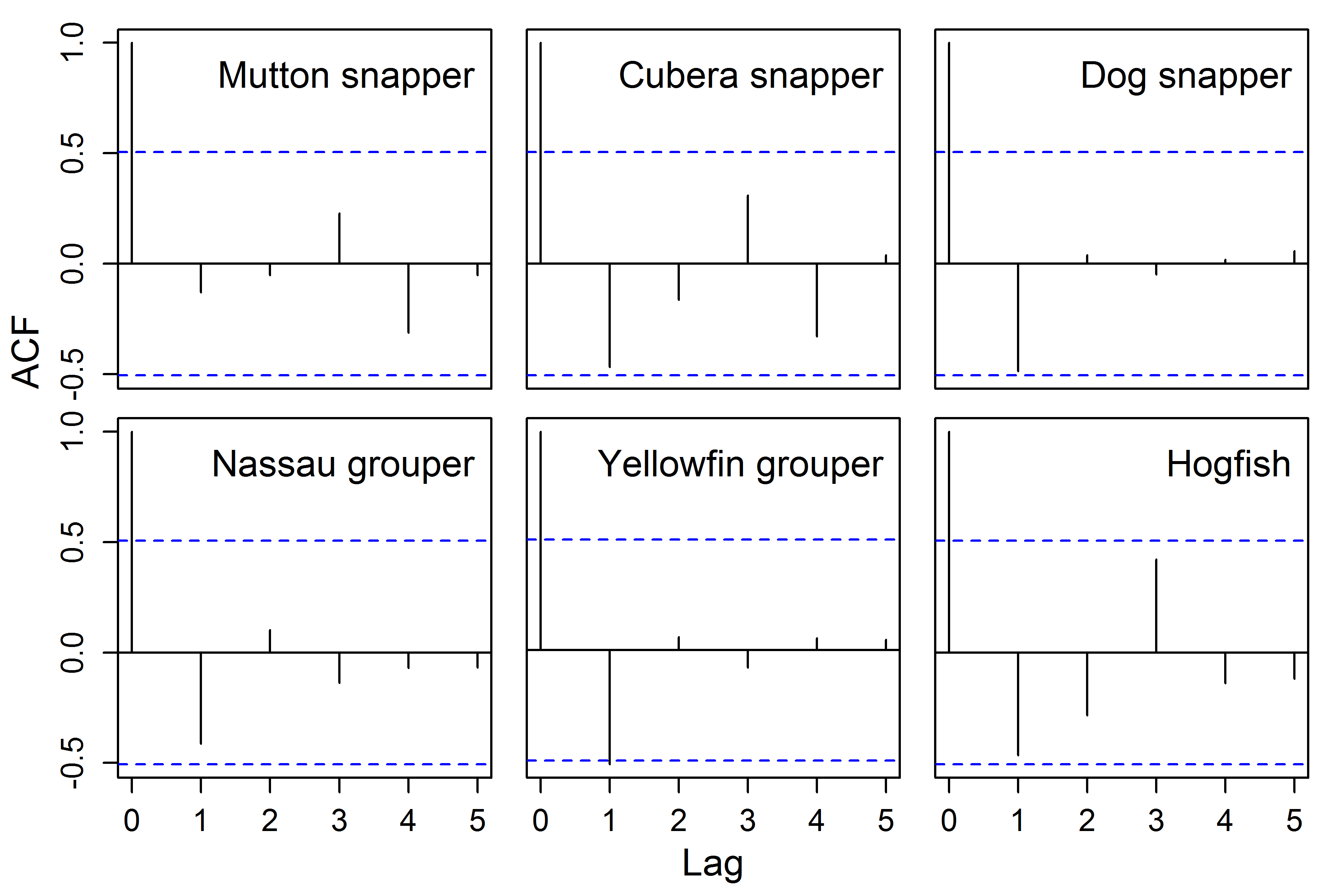

Supplement: Supplemental Information 7 — Autocorrelation function (ACF) values showing the lack of temporal correlation among five months (lag) for six trophy species in the reef crest habitat that showed significant interactions between zones and times based on the factorial ANOVA. [file peerj-02-274-s007.png]
